# Analysing the structure of antibodies using circular dichroism with an antibody augmented reference set and the algorithm SELCON

**DOI:** 10.1007/s00249-026-01828-5

**Published:** 2026-03-24

**Authors:** Søren Vrønning Hoffmann, Maria G. Bruque, Nykola C. Jones, Alison Rodger, Jean Aucamp, Tim R. Dafforn, Owen R. T. Thomas

**Affiliations:** 1 Department of Physics and Astronomy, ISA, Aarhus University, 8000 Aarhus C, Denmark; 2 School of Chemical Engineering, University of Birmingham, Edgbaston, B15 2TT Birmingham, UK; 3 School of Biosciences, University of Birmingham, Edgbaston, B15 2TT Birmingham, UK; 4 Research School of Chemistry , The Australian National University, ACT, 2601 Canberra, Australia; 5 Lonza Biologics, Slough, SL1 4DX UK

**Keywords:** Biotherapeutic proteins, Protein secondary structure analysis, Quality control, Therapeutic monoclonal antibodies (mAbs)

## Abstract

Proper folding of therapeutic monoclonal antibody (mAb) drugs is key to their efficacy and safety. Circular Dichroism (CD) spectroscopy is well suited for investigating the secondary structure of proteins, but the adequacy of the technique for antibody molecules has been severely impacted by the lack of suitable CD reference sets that include spectra of proteins of similar structure. Here we report on the performance of an expanded reference set that includes CD spectra of 14 mAbs, and uses structures derived from homology models. This enhanced reference set is part of a freely available Python-based software package, SSCalcPy-mAb, that includes the new SP-mAb178 reference set for secondary structure analysis using the SELCON3 method. We propose to employ both the secondary structure analysis results and a generated list of the proteins in the reference set that SELCON3 applies to derive the results that ascertain whether an antibody of interest is well-folded or not.

**Supplementary Information:**

The online version contains supplementary material available at 10.1007/s00249-026-01828-5.

## Introduction

Monoclonal antibodies (mAb) represent the cornerstone of the biopharmaceutical industry (Szkodny and Lee 2022; Kelley 2024; Chan et al. 2025). These hugely important modalities provide highly specific, targeted treatments for complex maladies and continue to lead biopharmaceuticals in numbers of approvals (Antibody Society Website) and sales (Saha et al. 2025) driven by the increasing global burden of cancers (Bizuayehu et al. 2024), autoimmune disorders (Miller 2023), and infectious diseases (Liu et al. 2025), rising R&D investments (Szkodny and Lee 2022; Kelley 2024; Malhotra et al. 2024; Chen et al. 2025), and advances in biosimilar and personalized therapies (Peeters et al. 2021; Jin et al. 2022; Walsh and Walsh 2022; Klein et al. 2024; Tsuchikama et al. 2024). Maintaining the correct 3D structure is vital for the efficacy and safety of biopharmaceutical proteins, (Sharma 2007; Vázquez‐Rey and Lang 2011; Makurvet 2021) but manufacturing environments pose significant challenges. For example, when mAbs are subjected to stress from low pH elution, viral-inactivation, and neutralization operations, structural changes and increased aggregation can occur; these are major issues complicating processing and reducing product yield. (Vázquez‐Rey and Lang, 2011; Roberts 2014; Mazzer et al. 2015). Analytical methods that can probe protein structure hierarchy in-process would significantly benefit the biopharmaceutical industry by improving the understanding of how manufacturing conditions impact mAb structure.

Circular dichroism (CD) is a sensitive non-destructive biophysical technique that is commonly employed for studying changes in the solution-state conformation of proteins (Kelly et al. 2005). CD is the difference in absorption of left and right circularly polarised light. For isotropic solution phase samples, CD reports on the helicity of transitions. In this paper we focus on the electronic transitions that occur in the backbone of proteins when light of wavelength 260 nm or shorter is incident on the sample. Since Moffit and Yang’s work on optical rotatory dispersion (ORD) of polypeptides (Moffitt and Yang 1956), it has been recognised that ORD and its absorption equivalent, CD, contain information about protein structure. This is because proteins have fairly well-defined secondary structure motifs including the α-helix and β-strand, and the chiroptical spectroscopy of the polypeptide backbone region has proved to be a source of structural information. In this work, the two types of peptide backbone transitions on which we focus are, to a reasonable approximation, (i) transitions of non-bonding orbitals to amide π* orbitals, which occur at about 220 nm, and (ii) π-π* transitions which occur at about 195 nm; though in an α-helix, coupling results in two bands, i.e., one at 208 nm and the other at 190 nm (Nordén et al. 2010; Bulheller et al. 2007).

A wide range of different methodologies have been developed to analyse CD spectra. We previously showed (Hall et al. 2013), using a ‘Leave-One-Out-Validation’ (LOOV) approach, that CDSSTR (Johnson 1999), SELCON3 (Sreerama and Woody 1993, 2000) and a self-organising map approach (Hall et al. 2013; Hall et al. 2014a; Hall et al. 2014b; Olamoyesan et al. 2021) gave equivalent fitting results when a high quality reference set is used. Arguably SELCON3 was slightly better for unusual structures than the other methodologies – in large part because of its higher quality constraints. In more recent work, (Hoffmann et al. 2025) we demonstrated the complementarity of infra-red (IR) absorbance and CD data, and produced a version of SELCON3 that can employ either CD or IR or a combination of both (IR-CD), which we validated using a LOOV approach on a slightly revised version of the SP175 reference set (Lees et al. 2006) combined with an IR reference set derived from work by Oberg et al. (2004).

Several software packages have been available for secondary structure analysis, including the original CDPro (Sreerama and Woody 2000), the web-based DichroWeb (Whitmore and Wallace 2004, 2008; Miles et al. 2022) and BeStSel (Micsonai et al. 2022), as well as the more recent Python based SSCalcPy (Hoffmann et al. 2025). CDPro features seven reference sets, which are also included in DichroWeb. Also available in DichroWeb are the much-improved SP175 (Lees et al. 2006) and SMP180 (Abdul-Gader et al. 2011) reference sets, with the latter incorporating membrane proteins. SSCalcPy includes revised versions of SP175 and SMP180 as described in detail in the Supplementary information in Hoffmann et al. (2025).

Common to all these CD structural analysis resources is that there has been little to no focus on analysis of monoclonal antibodies. In fact, only a single immunoglobulin G has been included in the SP175 reference set, and thus by extension also in SMP180. This may be viewed as a serious hindrance to CD spectroscopy’s use in the biopharmaceutical industry, particularly given the ongoing transformative impact of mAbs and other antibody therapeutics on this burgeoning sector (Lu et al. 2020; Peeters et al. 2021; Walsh and Walsh 2022; Jin et al. 2022; Qian et al. 2023; Kelley 2024; Klein et al. 2024; Tsuchikama et al. 2024). Because proper folding is closely related to the function of these biotherapeutic proteins, regulatory agencies mandate extensive characterisation of structure during biopharmaceutical development to ensure that the correct protein conformation is maintained throughout the manufacturing process and between batches (EMEA 1999, 2007; FDA 2015; Weiss et al. 2016). Therefore, enhancing the adequacy of CD spectroscopy for biopharmaceutical quality control is a pertinent task.

Here we report on a new development where the revised SP175 reference set has been augmented with 14 therapeutic monoclonal antibody CD spectra from Bruque et al. (2024) and implemented for mAb analysis using SELCON3 in an updated version of the Python code named SSCalcPy-mAb.

## Methods

The SELCON3 routine (Sreerama and Woody 2000) implemented and used in this work is part of the Python-based program and reference set package SSCalcPy, detailed by Hoffmann et al. (2025). The revised SP175 reference set (Lees et al. 2006) in the software was supplemented with a new set of 14 therapeutic monoclonal antibody CD spectra recorded down to 178 nm (Bruque et al. 2024). The new reference dataset, named SP-mAb178, is available at GitHub and included in SSCalcPy under AU-SRCD/SSCalcPy-mAb (Hoffmann and Jones 2025a) and ZENODO (Hoffmann and Jones 2025b). The secondary structure (SS) assignments of the 14 mAbs incorporated in the SP-mAb178 reference set are derived from antibody homology models (Bruque et al. 2024) using the DSSP method (Kabsch and Sander 1983) employing the structure grouping described in the supplementary information of Hoffmann et al. (2025). This yielded six secondary structure classes: ‘alpha regular’, ‘alpha distorted’, ‘beta regular’, ‘beta distorted’, ‘turns’, and ‘others’. Although the SSCalcPy software outputs results for these six structure classes, to prevent overinterpretation of the details of the SELCON3 secondary structure results, only four classes are presented here (Spencer and Rodger 2021). ‘Turns’ and ‘others’ remain the same, but ‘alpha regular’ and ‘alpha distorted’ are grouped into ‘helix’, while ‘beta regular’ and ‘beta distorted’ are defined as ‘sheets’. The SP175 reference set comprises 71 protein CD spectra. The SP-mAb178 reference set contains these same 71 spectra, plus additional spectra for the 14 therapeutic mAbs, which were designated mAbs 1 to 14 by Bruque et al. (2024). For this analysis, SP175 proteins are numbered 0–70, and the 14 mAbs —referenced at the end of SP175— are numbered 71–84, for a total of 85 reference spectra. As a note that will be important in the analysis presented below, SP175 already has one immunoglobulin G, a full mouse IgG (1IGT) (Harris et al. 1997), included as spectrum number 38. The addition of the mAbs to the reference set had no negative impact on the performance of SELCON3 (see supporting information S1).

To validate that SELCON3, when used with the new SP-mAb178 reference set, can correctly predict secondary structure content of mAbs, we performed a Leave-One-Out-Validation (LOOV). In this process, each of the mAbs 1–14 was, in turn, removed from SP-mAb178 and analysed with the remaining 84 reference spectra. The LOOV Python script is included in the SSCalcPy-mAb package under the folder “Tools”. In the present analysis, the secondary structure components are represented as fractions in the interval 0 to 1.

## Results and discussion

**Fig. 1:**
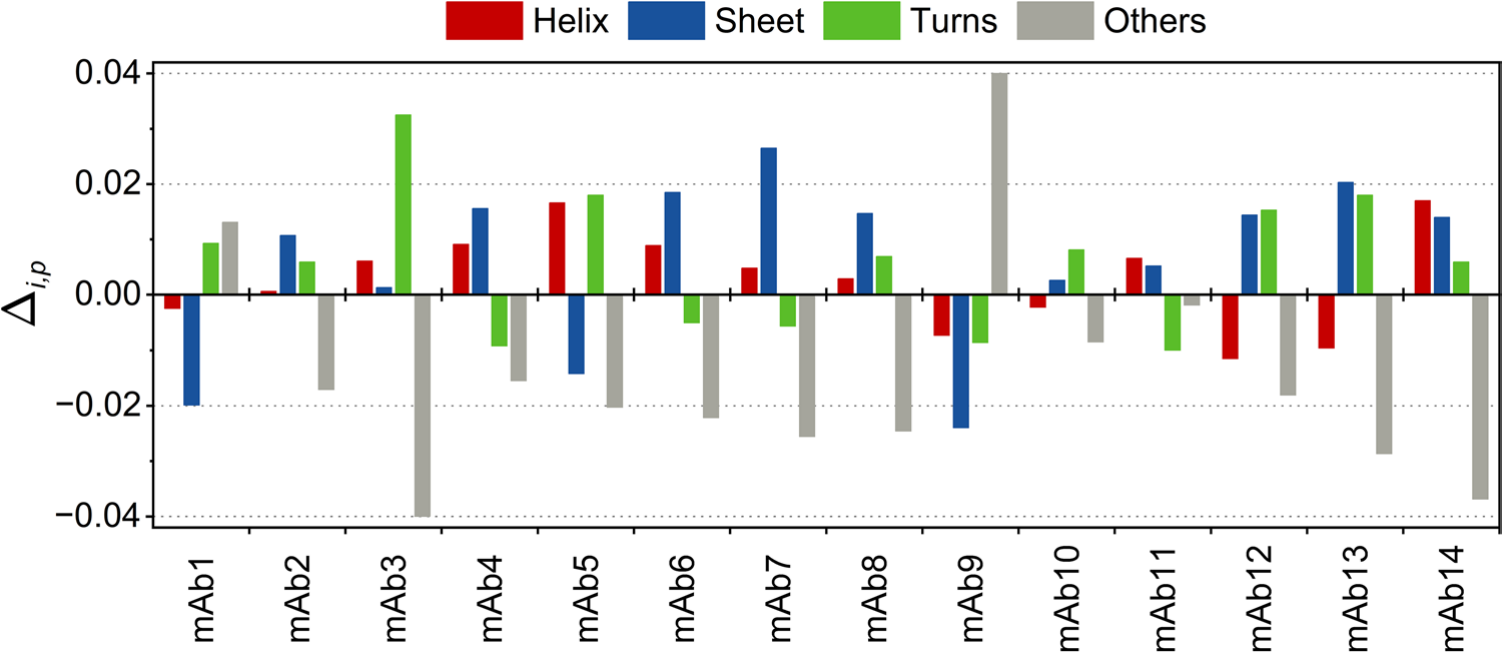
Results of LOOV analysis of the CD spectra of the 14 therapeutic mAbs with the SELCON3 routine, showing the differences (Δ_*i*,*p*_) between the DSSP analysis of their homology models and the SELCON3 results, for each of the 14 antibody proteins (*p*), and each of the four structural components (*i*)

The results of the LOOV analysis of the CD spectra of the 14 therapeutic mAbs analysed with the SELCON3 routine are presented in Fig. 1. For each of the 14 antibody proteins (*p*), and each of the four structural components (*i*), the difference between the secondary structure content derived from DSSP analysis of the homology models (Bruque et al. 2024), denoted ‘hSS_*i*,*p*_’, and the SELCON3 results denoted ‘SS_*i*,*p*___SELCON3_’, is shown. The original SELCON3 routine (Sreerama and Woody 2000) allows solutions where each secondary structure component is larger than –0.025 (–2.5%), permitting slightly negative values, and where the sum of all components is within 0.95–1.05 (± 5% from the ideal sum of 1). As the homology model secondary structure components always sum to 1, a normalisation of the SELCON3 results (nSS_*i*,*p*_) was performed so that the sum is also 1, i.e., nSS_*i*,*p*_ = SS_*i*,*p*___SELCON3_/Σ_*i*_ (SS_*i*,*p*___SELCON3_). It should be noted that none of the solutions contains problematic negative components, which would otherwise have compromised the normalization procedure. In the following, we denote the difference in each secondary structure component as Δ_*i*,*p*_ = hSS_*i*,*p*_ – nSS_*i*,*p*_. Here, Δ_*i*,*p*_ is calculated for each of the 14 mAbs in the LOOV analysis and is shown in Fig. 1.

The homology model and the SELCON3 analysis showed a maximum deviation of 0.04, with the larger deviations typically found in the ‘turns’ and ‘others’ secondary structure components. Notably, in many cases when the difference (Δ_i,p_) of one of these components is large, the corresponding Δ_*i*,*p*_ of the other typically has the opposite sign. The prediction of helix and sheets is typically much better, and the overall accuracy is quite satisfactory. To interrogate the outcome of the LOOV analysis further, we utilise two metrics (Hoffmann et al. 2025) for each secondary structure component, i.e.: first, the mean of absolute differences, mean(Δ_abs_,_*i*_) = Σ_*p*_ |Δ_*i*,*p*_|/n, summing over the 14 mAbs (*p*), and hence n = 14; and second, the standard deviations of the differences σ(Δ_*i*_). These metrics are shown in Table 1 for each of the secondary structure components.

**Table 1:** The performance of the secondary structure predictions for the 14 mAbs in the LOOV analysis using SELCON3

Metric	Helix	Sheets	Turns	Other
mean(Δ_abs_,_*i*_)	0.0076	0.0144	0.0113	0.0223
σ(Δ_*i*_)	0.0085	0.0149	0.0121	0.0200

The results for the mean of absolute differences, mean(Δ_abs_,_*i*_), reveal that for a given mAb SELCON3/SP-mAb178 correctly analyses the ‘helix’ structure content to better than 1%, and that for ‘sheets’ and ‘turns’ within 1.5%. The result for ‘others’ is slightly worse, but still better than 2.5%. Moreover, the standard deviations for each of the secondary structure components are quite small, and of nearly the same value as the mean absolute difference. Comparing the standard deviation with the individual results for each mAb in Fig. 1, the results are always found within ± 2 σ, as expected for a normal distribution. Therefore, both metrics show that the method is robust for all structural components, particularly so for ‘helix’, ‘sheets’ and ‘turns’.

To further analyse whether a mAb under investigation is correctly folded, the SELCON3 routine implemented in SSCalcPy-mAb offers information about which of the reference proteins were included in the algorithm’s calculation of the secondary structure fractions. CD spectra analysis routines such as CDSSTR (Johnson 1999) randomly select eight spectra from the reference set, check if the solution is valid, and repeats this process until up to 400 valid solutions are found, with the result presented as the average of the valid solutions. In contrast, the SELCON3 routine systematically compares the CD spectrum under investigation to all the reference spectra and sorts them, in descending likeness, i.e., increasing Root Means Square Deviations (RMSD). The routine starts by searching valid solutions from the three reference spectra with closest resemblance to the analysed spectrum and incrementally increases the number of reference spectra included in the analysis while searching for valid solutions. It is therefore possible to list the reference spectra of all the proteins included in valid solutions of the SELCON3 routine, and the list will be in descending order of likeness. The SELCON3 routine implementation in the SSCalcPy-mAb includes generation of this ordered list of names of used reference proteins in the output file.

Lists of the first 15 reference proteins employed in the LOOV analysis of each mAb in mAbs 1–14 are included in the supplementary information (S2) and Table 2 presents the results of LOOV analysis of mAbs 1–5. It should be noted that the total number of proteins from the SP-mAb178 reference set used by the algorithm varied from as few as 7 (mAb9) to all antibodies (mAbs 4, 5, 12, and 14). The former may be explained by inspection of the CD spectrum of mAb9 in Bruque et al. (2024). This reveals that the overall intensity of the spectrum is smaller compared to the other mAbs and it displays large spectral changes depending on pH. The overall similarity of its CD spectrum to other reference spectra in SP-mAb178 is therefore lower, resulting in fewer reference proteins used in the SELCON3 calculations. Conversely mAb9 is a less favoured match for other mAbs (Table 2 and S2).

**Table 2:** The order of the reference protein spectra (Ref prot1–15) of the SP-mAb178 reference set used to analyse mAb1–5, in order of descending likeness between the reference protein and the mAb spectrum under investigation

Spectra order	71: mAb1	72: mAb2	73: mAb3	74: mAb4	75: mAb5
Ref prot1	77: mAb7	75: mAb5	84: mAb14	38: IgG	76: mAb6
Ref prot2	74: mAb4	76: mAb6	78: mAb8	84: mAb14	78: mAb8
Ref prot3	81: mAb11	78: mAb8	81: mAb11	71: mAb1	84: mAb14
Ref prot4	84: mAb14	84: mAb14	71: mAb1	77: mAb7	72: mAb2
Ref prot5	38: IgG	80: mAb10	82: mAb12	76: mAb6	74: mAb4
Ref prot6	82: mAb12	73: mAb3	75: mAb5	83: mAb13	73: mAb3
Ref prot7	73: mAb3	38: IgG	38: IgG	78: mAb8	38: IgG
Ref prot8	80: mAb10	71: mAb1	74: mAb4	75: mAb5	80: mAb10
Ref prot9	76: mAb6	74: mAb4	77: mAb7	82: mAb12	83: mAb13
Ref prot10	78: mAb8	77: mAb7	76: mAb6	81: mAb11	71: mAb1
Ref prot11	75: mAb5	83: mAb13	72: mAb2	79: mAb9	77: mAb7
Ref prot12	79: mAb9	82: mAb12	83: mAb13	73: mAb3	82: mAb12
Ref prot13	72: mAb2	81: mAb11	80: mAb10	80: mAb10	81: mAb11
Ref prot14	83: mAb13	79: mAb9	79: mAb9	72: mAb2	79: mAb9
Ref prot15	17: Cerulo plasmin	14: Carbonic Anhydrase II	17: Cerulo plasmin	14: Carbonic Anhydrase II	14: Carbonic Anhydrase II

Table 2 shows that the SELCON3 analysis used the other 14 mAbs from the reference set for the most part, i.e., mAbs 1–14, excluding the mAb under investigation, and IgG (#38). In contrast, analysis of acid-stressed (pH 3) mAbs (detailed in supporting information S3), representative of misfolded species, shows frequent occurrences of several non-antibody proteins among their best matches. We therefore propose, in the context of biopharmaceutical quality control, that if analysis of an unknown mAb’s CD spectrum, using the SP-mAb178 reference set and the SELCON3 routine in the SSCalcPy-mAb software package, shows secondary structures consistent with that expected AND that the closest matches are to mAb1–14 + IgG, then the query mAb sample can be considered well-folded. It is generally important always to inspect the secondary structure solutions to make sure they are trustworthy. If the analysis only presents few and all identical SELCON3 solutions (i.e. zero deviation, see supporting information S3 for such an example), the secondary structure calculation is not reliable and should not be considered in support of a well-folded protein. This advice goes well beyond analysis of mAbs and should be applied for all proteins when investigating secondary structure using CD spectroscopy, using any reference set and method.

## Conclusions

The SP175 reference set has been augmented through the inclusion of CD spectra of 14 therapeutic mAbs to give a new reference set, SP-mAb178. This expanded dataset has been used with the SELCON3 routine in a LOOV analysis of mAbs 1–14, where it was found to give reliable calculations of secondary structure with good accuracy, such that on average results were within better than 1% for ‘helix’ and 1.5% for ‘sheets’ and ‘turns’. The implementation of the SELCON3 routine in the publicly available SSCalcPy-mAb software package, allows for further analysis through outputting a list of the best match between the mAb under investigation and the proteins in SP-mAb178 applied in the secondary structure analysis. We propose that both the calculated secondary structure, remembering to verify if the results are trustworthy, and the list of proteins generated are employed to judge whether the query mAb is well-folded, making this method a useful tool in quality control for these types of pharmaceuticals.

## Supplementary Information

Below is the link to the electronic supplementary material.

Supplementary file1 (PDF 229 kb)

## Data Availability

All data supporting the findings of this study are available within the paper, its Supplementary Information, and via the references in the paper to GitHub and Zenodo.
