# Supplementary material for: Analysing the structure of antibodies using circular dichroism with an antibody augmented reference set and the algorithm SELCON
Source: Eur Biophys J. 2026 Mar 24;55(3):419–25. doi: 10.1007/s00249-026-01828-5 (PMC13319872; doi:10.1007/s00249-026-01828-5)
Supplement: Supplementary file 1 — Supplementary file1 (PDF 229 kb) [file 249_2026_1828_MOESM1_ESM.pdf]

Supplementary information

Analysing the structure of antibodies using circular dichroism with an antibody augmented reference set and the algorithm SELCON

European Biophysical Journal

Søren Vrønning Hoffmann (ORCID: 0000-0002-8018-5433),[1] Maria G. Bruque (ORCID: 0009-0002-6191-8846),[2,3] Nykola C. Jones (ORCID: 0000-0002-4081-6405),[1] Alison Rodger (ORCID: 0000-0002-7111-3024),[4] Jean Aucamp (ORCID: 0009-0004-8574-6262),[5] Tim R. Dafforn (ORCID: 0000-0003-2257-6679)[3], Owen R. T. Thomas (ORCID: 0000-0001-8253-365X)[2]

[1]ISA, Department of Physics and Astronomy, Aarhus University, 8000 Aarhus C, Denmark

[2]School of Chemical Engineering and [3]School of Biosciences, University of Birmingham, Edgbaston, B15 2TT, U.K.

[4]Research School of Chemistry. The Australian National University, ACT, 2601, Australia

[5]Lonza Biologics, Slough, SL1 4DX, U.K.

Corresponding author: Søren Vrønning Hoffmann (vronning@phys.au.dk)

S1: Compared performance of SP175 and SP-mAb178 for common proteins

To ensure that the performance of SELCON3 analysis with the SP-mAb178 has not been compromised compared to SP175, we have analysed the difference in outcome of the two references sets for common proteins: myoglobin, concanavalin A and lysozyme. To this list, we have included an immunoglobulin G, to showcase the much-improved performance of the mAb augmented reference set.

Shown in Figure S1.1 is the difference between the SELCON3 secondary structure fractions (0 to 1) as the results using SP-mAb178 minus the results using SP175. For lysozyme and myoglobin, the changes are very small (<1%) and hence does not change the performance. For concanavalin A, there is a slight increase in 'sheet' and 'turns' structure at the expense of the 'other', which brings the 'sheet' content closer to the crystal structure. Interestingly, the number of solutions in the SELCON3 analysis of concanavalin A, which are finally averaged and presented as the result, was only 5 solutions when using the SP175 reference set. This can be compared to the 69 solutions for myoglobin. However, with the SP-mAb175 reference set, the number of solutions increased to 8 for concanavalin A. This shows that the addition of the monoclonal antibodies to the reference set improved the analysis quality of this sheet-rich protein.

For IgG, using SP-mAb178 clearly increased the ordered structures, and more strikingly, the number of SELCON3 solutions increased dramatically from just 4 to 73. In addition, the increase in 'helix' and the decrease in 'others' bring the results for all secondary structure components to within 1% of the crystal structure. Hence for IgG, while the SP175 reference set is not suitable for analysing antibodies, the new SP-mAb178 reference set clearly improves the quality of the analysis.

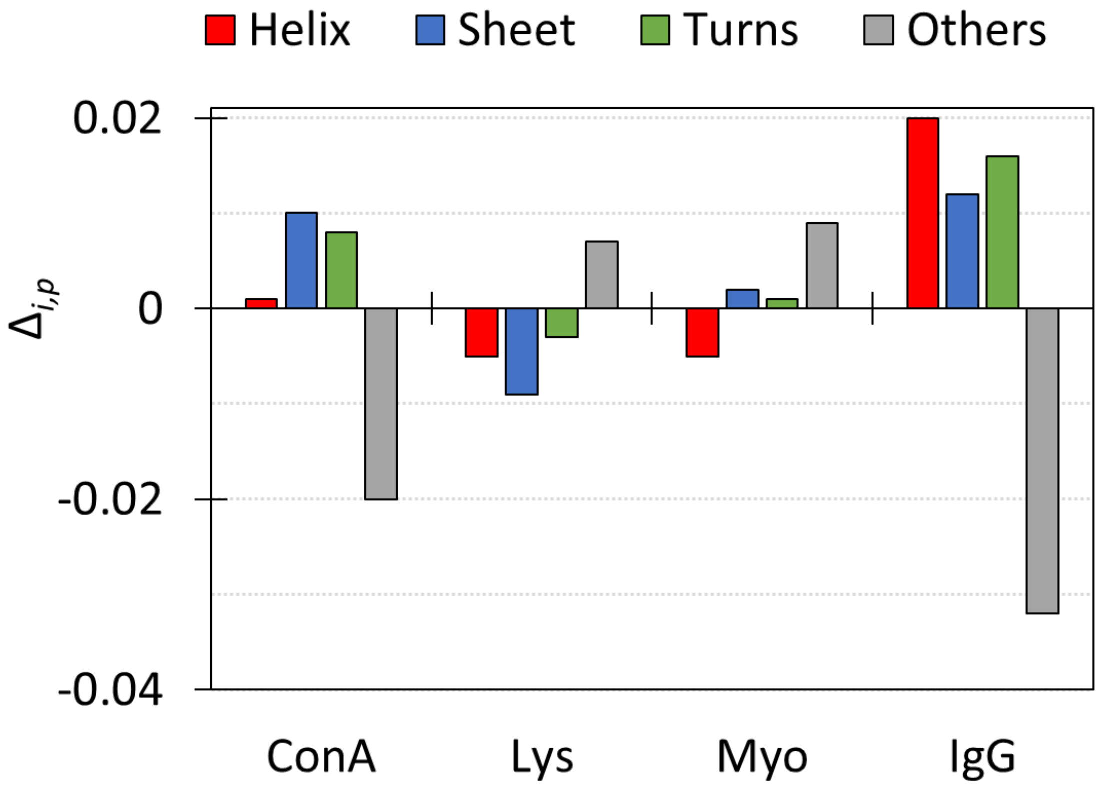

Fig. S1.1 The difference in secondary structure fractions calculated using SELCON3 with the reference sets SP-mAb178 and SP175. A positive value corresponds to an increase in the calculated fraction when using the SP-mAb178 reference set.

The improvement in analysing antibodies, besides the example of IgG above, may be further explored by making a full secondary structure analysis on all the mAb1-14 using only the revised SP175 reference set, i.e., where antibodies are only represented by IgG in the reference set.

Figure S1.2 shows the difference between the homology model secondary structures and the SELCON3 results using the pure SP175 reference set. When comparing these results with the LOOV analysis on the augmented

SP-mAb178 reference set shown in Figure 1 of the main paper, substantive deviations are noted. We therefore conclude that: (i) the SP175 reference set is not suitable for analysing antibodies; and (ii) the new SP-mAb178 reference constitutes a clear improvement in SELCON3 based secondary structure analysis for these biotherapeutic proteins.

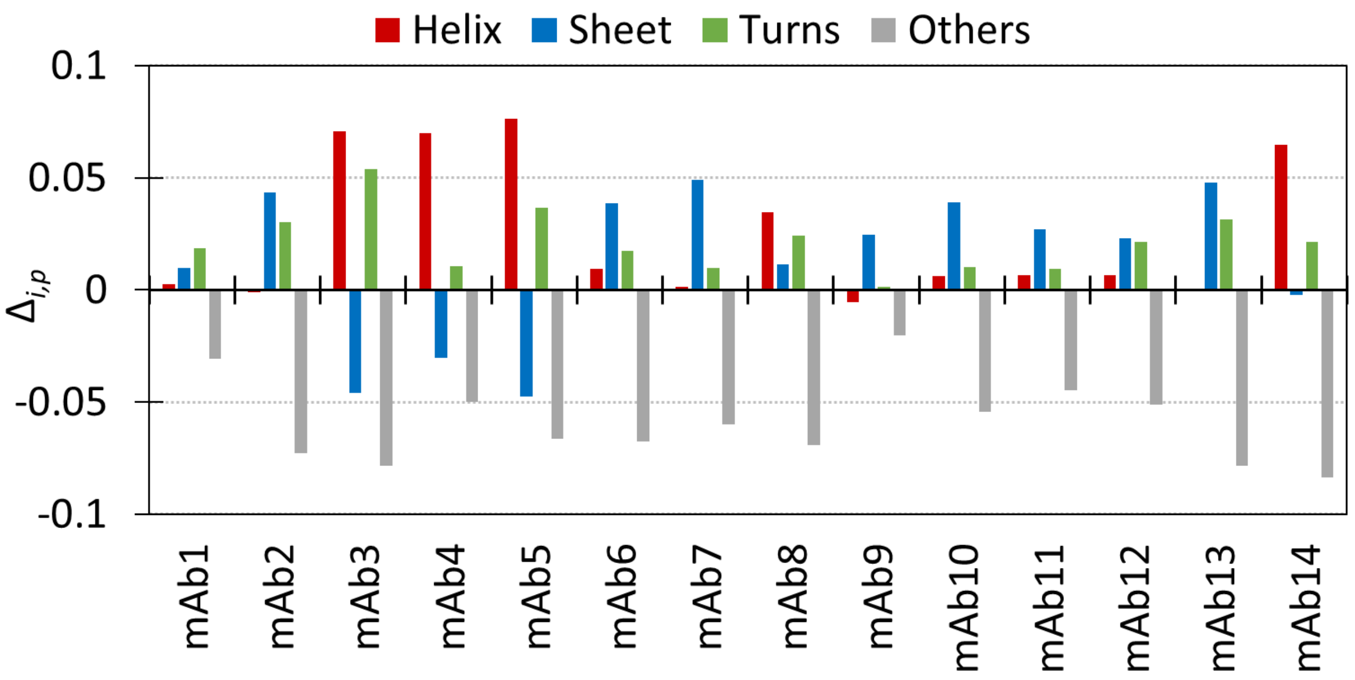

Fig. S1.2 Results of a pure SP175 analysis of the CD spectra of the 14 therapeutic mAbs with the SELCON3 routine, showing the differences (Δi,p) between the DSSP analysis of their homology models and the SELCON3 results, for each of the 14 antibody proteins (p), and each of the four structural components (i).

When increasing the size of a reference set it is prudent to ensure that predictive performance is not compromised. Here, the methodology employed by SELCON reduces this risk. Initially SELCON sorts the reference spectra according to similarity to the query proteins spectrum under analysis in terms of RMSD between them from low to high. In the algorithm, valid solutions are found starting from using only the 3 most alike reference spectra, and then adding more and more reference spectra, with decreasing similarity to the query spectrum, in the search for valid solutions. In the end, all valid solutions are averaged to yield the final SELCON secondary structure results. The SELCON routine therefore reduces the number of reference spectra used when performing the structure analysis.

To further demonstrate, beyond the examples of myoglobin, concanavalin A and lysozyme, that inclusions of mAbs into the SP175 does not compromise the SELCON3 performance, we undertook a full LOOV analysis of all the 71 proteins in SP175, one LOOV using the SP175 reference set and one LOOV using the augmented SP-mAb178 reference set. The standard deviation within the 71-reference data of the differences between the SELCON3 results and the crystal structures used in SP175, are essentially the same between the two LOOV analyses. The highest change in standard deviation is below 0.3% secondary structure, specifically Helix 0.14%, Sheet 0.25%, Turns 0.03% and Others 0.04%, demonstrating that the inclusion of mAbs1-14 does not diminish the quality of the SELCON3 analysis compared to the original SP175 reference set for non mAbs.

S2: List of the first 15 proteins used in mAb1-14 analysis

List of the first 15 proteins (Ref prot1-15) from the reference set SP-mAb178 used for each mAb1-14 in the LOOV analysis. The proteins in the reference set are numbered 0-70 for those in SP175 and 71-84 for the 14 therapeutic monoclonal antibodies (Bruque et al. 2024).

Spectra order	71: mAb1	72: mAb2	73: mAb3	74: mAb4	75: mAb5
**Ref prot1**	77: mAb7	75: mAb5	84: mAb14	38: IgG	76: mAb6
**Ref prot2**	74: mAb4	76: mAb6	78: mAb8	84: mAb14	78: mAb8
**Ref prot3**	81: mAb11	78: mAb8	81: mAb11	71: mAb1	84: mAb14
**Ref prot4**	84: mAb14	84: mAb14	71: mAb1	77: mAb7	72: mAb2
**Ref prot5**	38: IgG	80: mAb10	82: mAb12	76: mAb6	74: mAb4
**Ref prot6**	82: mAb12	73: mAb3	75: mAb5	83: mAb13	73: mAb3
**Ref prot7**	73: mAb3	38: IgG	38: IgG	78: mAb8	38: IgG
**Ref prot8**	80: mAb10	71: mAb1	74: mAb4	75: mAb5	80: mAb10
**Ref prot9**	76: mAb6	74: mAb4	77: mAb7	82: mAb12	83: mAb13
**Ref prot10**	78: mAb8	77: mAb7	76: mAb6	81: mAb11	71: mAb1
**Ref prot11**	75: mAb5	83: mAb13	72: mAb2	79: mAb9	77: mAb7
**Ref prot12**	79: mAb9	82: mAb12	83: mAb13	73: mAb3	82: mAb12
**Ref prot13**	72: mAb2	81: mAb11	80: mAb10	80: mAb10	81: mAb11
**Ref prot14**	83: mAb13	79: mAb9	79: mAb9	72: mAb2	79: mAb9
**Ref prot15**	17: Cerulo plasmin	14: Carbonic Anhydrase II	17: Cerulo plasmin	14: Carbonic Anhydrase II	14: Carbonic Anhydrase II

Spectra order	76: mAb6	77: mAb7	78: mAb8	79: mAb9	80: mAb10
**Ref prot1**	75: mAb5	71: mAb1	84: mAb14	77: mAb7	76: mAb6
**Ref prot2**	84: mAb14	82: mAb12	75: mAb5	74: mAb4	77: mAb7
**Ref prot3**	78: mAb8	81: mAb11	76: mAb6	71: mAb1	71: mAb1
**Ref prot4**	72: mAb2	38: IgG	73: mAb3	80: mAb10	72: mAb2
**Ref prot5**	80: mAb10	74: mAb4	72: mAb2	38: IgG	84: mAb14
**Ref prot6**	74: mAb4	84: mAb14	38: IgG	76: mAb6	75: mAb5
**Ref prot7**	38: IgG	80: mAb10	74: mAb4	84: mAb14	79: mAb9
**Ref prot8**	77: mAb7	79: mAb9	83: mAb13		78: mAb8
**Ref prot9**	71: mAb1	76: mAb6	82: mAb12		74: mAb4
**Ref prot10**	83: mAb13	78: mAb8	71: mAb1		38: IgG
**Ref prot11**	73: mAb3	73: mAb3	77: mAb7		82: mAb12
**Ref prot12**	79: mAb9	75: mAb5	80: mAb10		81: mAb11
**Ref prot13**	82: mAb12		81: mAb11		73: mAb3
**Ref prot14**	81: mAb11		79: mAb9		83: mAb13
**Ref prot15**	14: Carbonic Anhydrase II		14: Carbonic Anhydrase II		17: Ceruloplasmin

Spectra order	81: mAb11	82: mAb12	83: mAb13	84: mAb14
**Ref prot1**	82: mAb12	81: mAb11	74: mAb4	78: mAb8
**Ref prot2**	71: mAb1	77: mAb7	38: IgG	76: mAb6
**Ref prot3**	77: mAb7	71: mAb1	84: mAb14	74: mAb4
**Ref prot4**	73: mAb3	38: IgG	78: mAb8	38: IgG
**Ref prot5**	84: mAb14	84: mAb14	75: mAb5	75: mAb5
**Ref prot6**	38: IgG	73: mAb3	76: mAb6	71: mAb1
**Ref prot7**	74: mAb4	74: mAb4	73: mAb3	73: mAb3
**Ref prot8**	78: mAb8	78: mAb8	71: mAb1	77: mAb7
**Ref prot9**	76: mAb6	76: mAb6	14: Carbonic Anhydrase II	82: mAb12
**Ref prot10**	80: mAb10	80: mAb10	77: mAb7	72: mAb2
**Ref prot11**	75: mAb5	75: mAb5	82: mAb12	81: mAb11
**Ref prot12**	79: mAb9	79: mAb9	72: mAb2	80: mAb10
**Ref prot13**	72: mAb2	83: mAb13	80: mAb10	83: mAb13
**Ref prot14**	83: mAb13	72: mAb2		79: mAb9
**Ref prot15**	17: Cerulo plasmin	17: Cerulo plasmin		14: Carbonic Anhydrase II

S3: Identification of misfolded mAbs

Following the conclusion in the main text of this paper, where we propose that both the calculated secondary structure and the list of proteins generated is employed to judge whether the query mAb is well-folded, we have further analysed what output may be expected when a mAb protein under investigation is misfolded. For this analysis, we have used the CD spectra of acid-stressed (pH 3) mAbs from Bruque et al. (2024).

An investigation of Figure 1 in Bruque et al. (2024), where the CD spectra of mAbs1-14 recorded at pH 3 and neutral pH are compared, shows that the low pH spectra of mAbs 7, 8, 9, 11, and to some extent 14, are quite different from their neutral pH counterparts. The SELCON3 analysis of these low pH spectra, using the SP-mAb178 reference set, used the reference proteins shown in the Table immediately below.

Spectra order	mAb7 pH 3	mAb8 pH 3	mAb9 pH 3	mAb11 pH 3	mAb14 pH 3
**Ref prot1**	17: **Ceruloplasmin**	54: **Pepsinogen**	67: **Superoxide Dismutase [Cu-Zn]**	79: mAb9	79: mAb9
**Ref prot2**	79: mAb9	17: **Ceruloplasmin**	4: **alpha-chymotrypsin**	17: **Ceruloplasmin**	74: mAb4
**Ref prot3**	54: **Pepsinogen**	79: mAb9	30: **Elastase**	54: **Pepsinogen**	77: mAb7
**Ref prot4**	38: IgG	67: **Superoxide Dismutase [Cu-Zn]**	5: **alpha-chymotrypsinogen**	38: IgG	38: IgG
**Ref prot5**	74: mAb4	14: **Carbonic Anhydrase II**	54: **Pepsinogen**	74: mAb4	71: mAb1
**Ref prot6**	14: **Carbonic Anhydrase II**	38: IgG	14: **Carbonic Anhydrase II**	77: mAb7	82: mAb12
**Ref prot7**	77: mAb7	74: mAb4	63: **Rubredoxin**	82: mAb12	84: mAb14
**Ref prot8**	82: mAb12	3: **alpha-bungarotoxin**	45: **Monellin**	71: mAb1	
**Ref prot9**	71: mAb1	77: mAb7		14: **Carbonic Anhydrase II**	
**Ref prot10**	3: **alpha-bungarotoxin**	82: mAb12		3: **alpha-bungarotoxin**	
**Ref prot11**	83: mAb13	71: mAb1		81: mAb11	
**Ref prot12**	81: mAb11	81: mAb11		84: mAb14	
**Ref prot13**	84: mAb14	83: mAb13		83: mAb13	
**Ref prot14**	76: mAb6	84: mAb14			
**Ref prot15**	80: mAb10	76: mAb6			

The results for mAb7, 8, 9 and 11 clearly show that at least four non-mAbs proteins are used for the analysis; the most extreme case is for mAb9, where none of the mAb1-14 references are used. The list of reference proteins employed for the analysis therefore clearly indicates that these mAbs are misfolded compared to the neutral pH mAbs used in the reference set (see Table 2 and S2).

The result for mAb14 is not that clear. Although only very few reference proteins are used in the analysis, all of them are mAbs. It should be noted that among the spectra selected for this analysis, mAb14 is the mAb at pH 3 which has the closest resemblance to the neutral pH CD spectrum and hence is a clue as to why only mAbs reference spectra were used. However, this also shows that further clarification of the SELCON3 results need to be considered when judging the validity of the analysis. The SSCalcPy output for mAb14 is shown in the table below:

Helix	Sheet	Turns	Other	Sum	H Dev	S Dev	T Dev	Ot Dev	selcon?	#solu	RMSD
6.5	46.7	10.3	35.3	98.7	0.0	0.0	0.0	0.0	Selcon3	5	0.000

Helix, Sheet, Turns, and Other refer to the secondary structure components results expressed as percentages. The H Dev, S Dev, T Dev, and Ot Dev are the respective standard deviations of these structure components taken between the 5 (denoted #solu) SELCON3 (denoted selcon?) solutions. Finally, the RMSD is the root mean square deviation between the SELCON3 refitted spectrum and the spectrum under investigation (mAb14 pH 3).

The fact that very few solutions (5) are found and that the refitted spectrum is fully identical to the CD spectrum under investigation (RMSD = 0.000), are clear signs that the SELCON3 routine has failed. Additionally, the

standard deviations between the solutions are 0.0, i.e., the 5 solutions are, in fact, all identical. This clearly shows that no reliable SELCON3 solution is found.

This constitutes an example of the care that generally should be put into what SELCON3 results are trustworthy. Few solutions, all of which are identical (zero deviation) and a “perfectly” refitted spectrum must be taken as a clear sign of a failed analysis. This advice goes well beyond analysis of mAbs and should be generally applied to any CD based secondary structure analysis.
